# Patient-Reported Outcomes of Microfracture, Nanofracture, and K-Wire Drilling in Talus Osteochondral Lesions

**DOI:** 10.3390/diagnostics15172255

**Published:** 2025-09-06

**Authors:** Ahmet Görkem Kasapoğlu, Mehmet Arıcan, Yıldıray Tekçe, Giray Tekçe, İlyas Kaban

**Affiliations:** 1 Orthopedics and Traumatology Department, Afyonkarahisar State Hospital, 03100 Afyonkarahisar, Turkey; gorkemkasapoglu@yahoo.com; 2 Orthopedics and Traumatology Department, Medical Faculty, Duzce University, 81000 Düzce, Turkey; 3 Orthopedics and Traumatology Department, Gebze Fatih State Hospital, 41400 Kocaeli, Turkey; yildiraytekce@gmail.com (Y.T.); yarig2906@gmail.com (G.T.); 4 Orthopedics and Traumatology Department, Duzce Atatürk State Hospital, 81000 Düzce, Turkey; drilyaskaban@gmail.com

**Keywords:** talus, osteochondral lesion, microfracture, nanofracture, K-wire drilling

## Abstract

**Background/Objectives:** Different patient-reported outcomes and radiological results are reported depending on whether microfracture, drilling, or nanofracture is utilized in the arthroscopic treatment of talus osteochondral lesions, but the first-line treatment is still controversial. The aim of this study is to evaluate the early patient-reported outcomes of microfracture, nanofracture, and antegrade drilling methods in talus anteromedial osteochondral lesions. **Methods:** A total of 77 patients who presented with ankle pain between October 2016 and June 2022, were diagnosed with talus osteochondral lesions, and underwent microfracture (*n*: 27), nanofracture (*n*: 25), and K-wire drilling (*n*: 25) were included. Demographic data of the patients were evaluated, such as age, gender, lesion side, dominant extremity, body mass index (BMI), smoking status, smoking (pack/day-year), and symptom duration. Patient-reported outcomes of the patients were evaluated with VAS (visual analog scale) and AOFAS (American Orthopedic Foot & Ankle Society) scores measured before surgery and at 6 and 12 months after surgery. The results were evaluated at the significance level of *p* < 0.05. **Results:** There were no statistically significant differences among the microfracture, nanofracture, and drilling groups in terms of age, gender, lesion side, dominant extremity, BMI, smoking, or daily cigarette use (*p* = 0.121, *p* = 0.852, *p* = 0.956, *p* = 0.731, *p* = 0.881, *p* = 0.769, *p* = 0.124). Similarly, the mean duration of symptoms did not differ significantly between the groups (*p* = 0.336). Although AOFAS and VAS scores significantly improved in all groups (*p* = 0.0001), there were no statistically significant differences between the microfracture, nanofracture, and drilling groups at preoperative, 6th-, and 12th-month measuring points. The microfracture group showed a significantly higher AOFAS improvement from preop to 6 months compared to the other groups (*p* = 0.012), though no differences were found between nanofracture and drilling or in 12-month changes. VAS percentage changes showed no significant differences among groups at either time point. **Conclusions:** All treatment groups had similar baseline characteristics and outcomes, with the microfracture group showing a greater functional improvement at 6 months.

## 1. Introduction

Conservative treatment methods for talus osteochondral lesions, such as rest, activity modification, immobilization, pain and inflammation control, and physical therapy and rehabilitation, are generally preferred for small and stable lesions, in the early stages, and when symptoms are mild. If significant improvement is not observed within 3–6 months despite conservative treatment, or if the lesion progresses, surgical procedures (arthroscopic bone marrow stimulation methods, mosaicplasty, retrograde drilling, osteochondral transplantation) should be performed [1]. Arthroscopic bone marrow stimulation methods are still an effective treatment method for talus osteochondral lesions, especially lesions smaller than 150 mm^2^ [1,2]. One of the bone marrow stimulation methods, the microfracture technique, has been used frequently from past to present; however, the use of other techniques such as nanofracture and drilling methods has increased in the last 20 years [3,4,5]. Considering the relatively high incidence of osteochondral lesions of the talus, especially in young adult patients, and the fact that some of these lesions are not detected until the time of arthroscopy, the importance of applying a single-stage cartilage surgical procedure such as bone marrow stimulation methods is quite high [6]. In bone marrow stimulation methods, in cases of problems such as access to mesenchymal stromal cells and subchondral bone structure/overgrowth, the creation of vertical walls during defect preparation, increasing the depth of subchondral penetration, using a smaller awl diameter, and increasing the number of subchondral perforations have become very important [7,8]. In the arthroscopic treatment of talus osteochondral lesions, different clinical and radiological results are reported with microfracture, drilling, or nanofracture, and the gold-standard treatment is still controversial [4,9,10,11]. Choi et al. evaluated a total of 90 patients who underwent arthroscopic subchondral drilling (*n*: 40) and microfracture (*n*: 50) in their talus osteochondral lesions, and they reported similar clinical results with a mean follow-up period of 43 months [10]. In another study, Kraeutler et al. compared microfracture and drilling in articular cartilage lesions, and they emphasized that the drilling method has better biological properties than microfracture, as well as provides less damage to the subchondral bone and more access to the marrow stroma. In addition, it was stated in this study that the number of studies comparing microfracture and drilling for focal cartilage defects was insufficient in the literature [11]. There is still controversy regarding the non-standardized parameters of bone marrow stimulation techniques, such as canal diameter, depth, and access to subchondral tissue [12,13]. In the cadaver study by Gianakos et al., who evaluated the morphological changes, microarchitectural structure, and degree of access to subchondral bone marrow after the application of different bone marrow stimulation techniques in talus osteochondral lesions using microcomputed tomography (μCT) analysis with a 1.00 mm microfracture awl, 2.00 mm standard microfracture awl, and 1.25 mm Kirschner wire (K-wire) drilling, it was stated that the bone marrow stimulation method performed with larger-diameter hand tools caused increased trabecular compression and sclerosis in the regions adjacent to the defect [12]. In the clinical case study by Benthien et al. comparing the microfracture and nanofracture in cartilage lesions, it was stated that the nanofracture facilitates access to the subchondral bone marrow, while the needle size makes drilling the subchondral bone easier and more predictable. It was also emphasized that the clinical results were comparable to a microfracture [13]. When the literature was examined, although there were clinical studies in which microfracture, nanofracture, and drilling techniques were applied separately in talus osteochondral lesions, no study was found that compared all three techniques clinically. The purpose of the present study was to evaluate the early patient-reported outcomes of microfracture, nanofracture, and K-wire drilling techniques in talus anteromedial osteochondral lesions.

## 2. Materials and Methods

Between October 2016 and June 2022, 83 patients who applied to the Orthopedics and Traumatology clinic with ankle pain complaints and were diagnosed with a talus osteochondral lesion or radiologically detected anteromedial full-thickness cartilage lesion, who were aged between 18 and 55 years, who underwent microfracture, nanofracture, and antegrade K-wire drilling, and who met the inclusion criteria were included in this study, and the patient-reported outcomes were evaluated retrospectively. This study was approved by the Düzce University Faculty of Medicine non-invasive health research ethics committee (2023/118) and all patients were informed about the study, with an informed consent form and ethical approval obtained.

Patients aged between 18 and 55 years, with isolated anteromedial talar dome osteochondral lesions, with symptoms for more than 6 months, with at least 12 months of follow-up, with lesions smaller than 2 cm^2^, and with lesions on one extremity were included in this study. Patients aged under 18 or over 55 years, with lesions larger than 2 cm^2^, with corticosteroid, platelet-rich plasma, hyaluronic acid, etc., injections to the ankle within the last 6 months, and with a previous ankle surgery in the ankle region were excluded from the study. A total of 77 patients who met the inclusion criteria [microfracture (*n*: 27), nanofracture (*n*: 25), drilling (*n*: 25)] were included in this study. According to our criteria, patients were excluded due to age (*n* = 5) and hyaluronic acid injection in the last 6 months (*n* = 1).

Demographic data of the patients were evaluated as follows: age, gender, lesion side, dominant extremity, BMI, smoking status, cigarettes (pack/day-year), and symptom duration. Patient-reported outcomes of the patients were evaluated with VAS and AOFAS scores measured before surgery and at 6 and 12 months after surgery. The AOFAS hindfoot score consists of 9 questions that evaluate pain, function, and alignment; 1 question is about pain, 7 questions are about function, and 1 question is about alignment. Patients are evaluated out of 100 points in total, as the pain section has 40 points, function section has 50 points, and alignment section has 10 points. A high score indicates a good result. The VAS can be used for measuring pain, which is somewhat difficult to quantify objectively. The simplest type of VAS is a straight horizontal line, typically 10 cm in length. Assessment of patient outcomes was performed by the author (İK), who was not involved in the surgical treatment procedures to ensure minimal bias.

**Figure 1:**
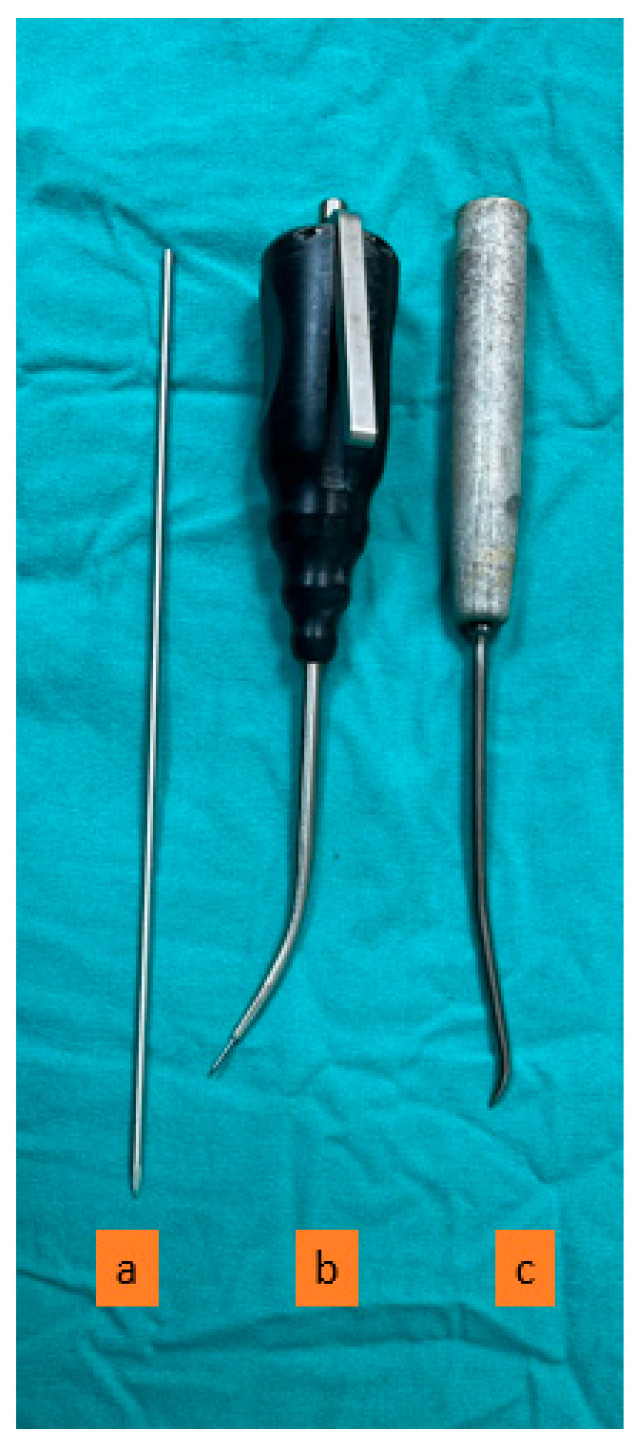
Arthroscopic bone marrow stimulation hand tools: (**a**) K-wire, (**b**) nanofracture, (**c**) microfracture.

**Figure 2:**
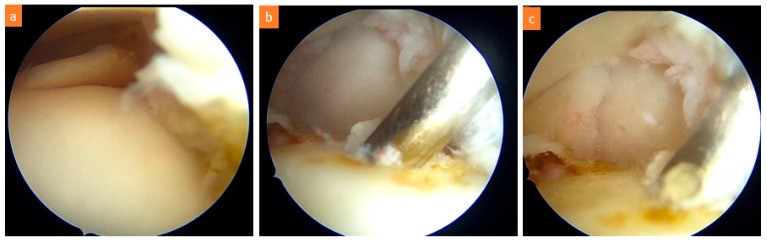
(**a**) Arthroscopic ankle image of a 32-year-old female patient with anteromedial talus osteochondral lesion, (**b**) bone marrow stimulation method with 1.2 mm K-wire, (**c**) release of fatty droplet indicating sufficient depth reached.

Arthroscopic procedures were performed under general anesthesia, in the supine position, using standard anteromedial and anterolateral portals. A marked probe was inserted through the anteromedial portal to elevate the unstable cartilage from the base, and the stability of the lesion and cartilage continuity were determined. An arthroscopic shaver was used to debride the unstable cartilage flaps. The lesion size was measured using the marked probe. Microfracture, nanofracture, and K-wire drilling hand tools were used to create several small holes in the subchondral bone through the anteromedial portal (Figure 1). The adequacy of the hole depth was determined by the release of fatty droplets (Figure 2).

All patients followed the same postoperative physical therapy protocol after undergoing arthroscopic bone marrow stimulation for anteromedial osteochondral lesions of the talus. Cold compresses were applied for 20 min, five times daily, for the first two weeks. Beginning on the first postoperative day, patients performed active range-of-motion exercises, including plantar flexion, dorsiflexion, abduction, adduction, and rotation, without weight bearing. Weight bearing was restricted for the first four weeks. Starting at the end of the first postoperative month, patients were gradually allowed to bear weight, and progressive physiotherapy was continued accordingly.

### Statistical Analysis

The statistical analyses in this study were performed using NCSS (Number Cruncher Statistical System) 2007 Statistical Software (Kaysville, UT, USA). In addition to descriptive statistical methods (mean, standard deviation, median, interquartile range), the distribution of the variables was assessed using the Shapiro–Wilk normality test. For normally distributed variables, paired one-way analysis of variance (ANOVA) was used for within-group (time-based) comparisons, and a Newman–Keuls multiple comparison test was applied for post hoc subgroup analyses. For comparisons between independent groups, one-way ANOVA was used, followed by Tukey’s post hoc test for subgroup comparisons. For variables that did not follow a normal distribution, the Kruskal–Wallis test was employed for intergroup comparisons, and Dunn’s multiple comparison test was used for post hoc subgroup analyses. For categorical (qualitative) variables, the chi-square test was used. All results were evaluated at a significance level of *p* < 0.05.

## 3. Results

When the age, gender, lesion side, and dominant extremity distributions of the microfracture, nanofracture, and drilling groups were compared, no statistically significant difference was observed (*p* = 0.121, *p* = 0.852, *p* = 0.956, *p* = 0.731). No statistically significant difference was observed between the BMI, smoking, and daily number of cigarettes (pack/day/year) when reviewing the averages of the microfracture, nanofracture, and drilling groups (*p* = 0.881, *p* = 0.769, *p* = 0.124). Additionally, no statistically significant difference was observed between the mean durations of symptoms (months) in all three groups (*p* = 0.336) (Table 1).

**Figure 3:**
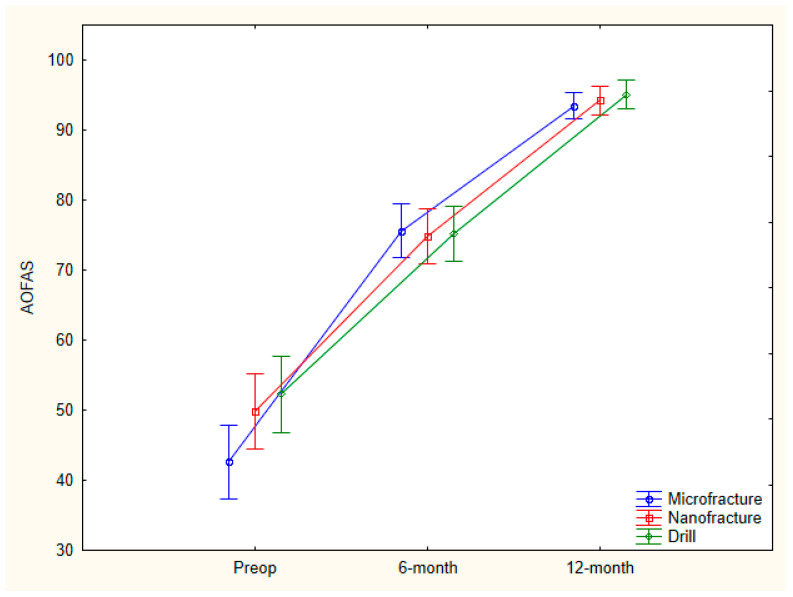
Comparison of AOFAS scores (preop, 6 and 12 months) of microfracture, nanofracture, and K-wire drilling groups.

**Figure 4:**
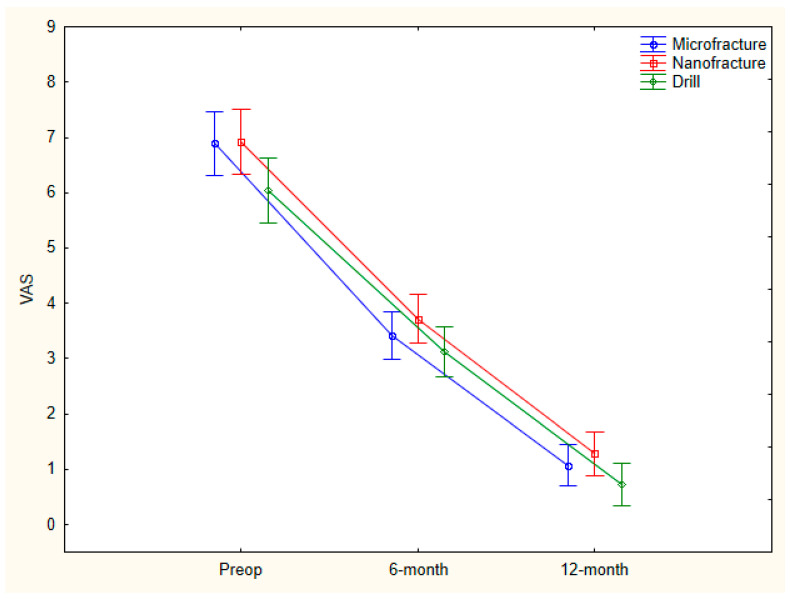
Comparison of VAS scores (preop, 6 and 12 months) of microfracture, nanofracture, and K-wire drilling groups.

Although AOFAS values showed a statistically significant increase (*p* = 0.0001) in all groups during the study period, no statistically significant difference was observed between the preoperative, 6th-month, and 12th-month AOFAS averages of the microfracture, nanofracture, and drilling groups (*p* = 0.053, *p* = 0.956, *p* = 0.528). In addition, although VAS values among all groups showed a statistically significant increase (*p* = 0.0001) during the study period, no statistically significant difference was observed between the preoperative, 6th-, and 12th-month VAS averages of the microfracture, nanofracture, and drilling groups (*p* = 0.066, *p* = 0.178, *p* = 0.127) (Table 2) (Figure 3 and Figure 4).

There was a statistically significant difference the AOFAS preoperative–6th month percentage (%) change mean score between the microfracture, nanofracture, and drilling groups (*p* = 0.012).

The AOFAS preoperative–6th month percentage change mean score of the microfracture group was found to be statistically significantly higher than those of the nanofracture and drilling groups (*p* = 0.047, *p* = 0.015), and there was no statistically significant difference between the AOFAS preoperative–6th month percentage change mean scores of the nanofracture and drilling groups (*p* = 0.850). There was also no statistically significant difference between the AOFAS preoperative–12th month percentage change mean scores of the microfracture, nanofracture, and drilling groups (*p* = 0.734).

No statistically significant difference was observed between the preoperative–6th month and preoperative–12th month VAS percentage change mean scores among the microfracture, nanofracture, and drilling groups (*p* = 0.458, *p* = 0.316) (Table 3).

## 4. Discussion

One of the current surgical methods for the treatment of talus osteochondral lesions is bone marrow stimulation techniques, which serve to stimulate blood flow from the subchondral bone tissue to the site of damage [14,15]. The classical microfracture technique has long been used as the first treatment option among bone marrow stimulation techniques in osteochondral cartilage lesions due to its advantages, such as cost, ease of application, and also its positive clinical results shown in the literature in a sports/active patient group [16,17]. Due to some negative aspects (poor results for larger defects >2 cm × 2 cm, does not address bone defects, requires limitation of weight bearing for 6–8 weeks) encountered with the widespread use of microfracture, other bone marrow stimulation methods such as debridement, abrasion arthroplasty, and subchondral drilling (K-wire or a drill) have begun to be used frequently in the literature [13,18]. In addition, in recent years, nanofracture, a new bone marrow stimulation method with a smaller diameter, the capacity to create a greater number of subchondral holes, and the ability to penetrate deeper into the subchondral bone, has taken its place among these treatments [4,19].

In our clinical study, where we evaluated the patient-reported outcomes of patients who underwent microfracture, nanofracture, and K-wire antegrade drilling in talus anteromedial osteochondral lesions retrospectively, although the AOFAS score showed a faster recovery after microfracture in the early period, there was no significant difference between the 6th- and 12th-month VAS and AOFAS scores of all three groups.

The mechanical differences in the drilling instruments used in the subchondral bone may create different responses for the subchondral bone architecture [3,20]. Warren et al. evaluated different bone marrow stimulation techniques and the resulting fluid permeability of the subchondral bone, along with radiopaque contrast material flow in a human talus bone model, using μCT image analysis and 3D modeling. They concluded that the nanofracture technique showed significantly improved fluid permeability throughout the surrounding trabecular structure compared to microfracture and K-wire drilling techniques. It was stated that the microfracture technique allowed some fluid flow, but only to the area immediately surrounding the fracture site. It was emphasized that the K-wire drilling technique allowed very little fluid flow. It was stated that the nanofracture technique should be the preferred method of subchondral bone preparation for osteochondral lesions of the talus [3]. Similarly, Walsh et al. compared microfracture, 1–2 mm K-wires, a 1 mm drill, and nanofracture in a sheep model for accessing bone marrow in cartilage defects. It was stated that nanofracture obtained thin, fragmented cancellous bone channels without rotational heat generation. In addition, it was emphasized that nanofracture showed superior bone marrow access with multiple trabecular access channels extending 9 mm into the subchondral bone compared to microfracture, drilling, and K-wire stimulation [20]. New areas of research have emerged with the better understanding of the importance of the interaction between articular cartilage and subchondral bone, such as channel formation, depth, cell requirement, and the quantity and quality of the formed cartilage [3,12,21]. There are studies in the literature in which only one method is used or two methods are compared for bone marrow stimulation methods such as microfracture, K-wire drilling, and nanofracture used in the treatment of osteochondral lesions of the talus, but no study comparing three groups as we did has been found. In an animal experiment model conducted by Zedde et al., a full-thickness osteochondral lesion was created in the femoral condyles of sheep knees, where one condyle was treated with microfracture and the other condyle with nanofracture then followed for 6 months. When evaluated later with μCT, it was emphasized that the nanofracture had deeper penetration and a smaller diameter and disrupted the subchondral bone microarchitecture less, cyst formation was less, it caused less trabeculation, and it was a more innovative method compared to the microfracture [22].

In a clinical study, Choi et al. performed a microfracture on 50 patients with small or medium-sized talus osteochondral lesions and drilling on 40 patients. After surgery, AOFAS, VAS, and AAS (ankle activity score) were evaluated, and in an average follow-up period of 43 months, all scores of both methods showed statistically significant improvements, and it was stated that neither method was superior to the other. They emphasized that both can be used safely for medium-sized lesions [10]. In another study by Kasman et al. on 28 patients with talus osteochondral lesions (<1.5 cm^2^), they compared drilling and microfracture for small–medium-diameter lesions and reported similar results to Choi [23]. This study, which is similar to our study in terms of patient number, follow-up period, and epidemiological characteristics, supports our clinical results. The fact that the nanofracture technique was also applied in addition to these two methods is one of the positive aspects of our study, and additional clinical studies comparing all three techniques are needed. In contrast to these results, in a clinical study conducted by Polat et al. on 82 patients, they compared the clinical results of at least 5 years of microfracture and debridement treatment of ankle talus osteochondral lesions using AOFAS and VAS. It was emphasized that both debridement and microfracture were significantly beneficial in pathologies with a lesion size of 1.7 ± 0.7 cm^2^ [24].

When we examined the literature, we found that nanofracture, which has become popular in the treatment of talus osteochondral lesions in recent years, has been evaluated in only a few studies. Tahta et al.’s study comparing nanofracture and hyaluronic acid-based acellular scaffolds with the concentration of autologous bone marrow aspirate in talus osteochondral lesions has indicated that a nanofracture is beneficial in talus osteochondral lesions [4]. In our study, although the AOFAS score improvements in the first 6 months were found to be statistically significantly different in the microfracture group compared to nanofracture and drilling, no statistically significant difference was found in the final controls.

When the limitations of our study, in which we applied microfracture, nanofracture, and anterograde K-wire drilling in talus anteromedial osteochondral lesions, were evaluated, first of all, it was noted that our study was a retrospective study. The second limitation was the short follow-up period and the small number of patients in each group. However, we think that it is also valuable to be the first comparative clinical study of all three techniques in talus osteochondral lesions.

## 5. Conclusions

Microfracture, nanofracture, and antegrade K-wire drilling techniques have all demonstrated significant improvements in ankle pain and function in patients with small-sized anteromedial osteochondral lesions of the talus. Among these, early AOFAS (American Orthopaedic Foot & Ankle Society) scores were highest in patients treated with the microfracture technique compared to those treated with nanofracture or K-wire drilling. However, to accurately assess the individual efficacy of each technique, further prospective clinical studies with larger patient cohorts and long-term follow-up are necessary.

## Figures and Tables

**Table 1:** Demographic characteristics of microfracture, nanofracture, and K-wire drilling groups.

		Microfracture (*n*: 27)		Nanofracture (*n*: 25)		Drilling (*n*: 25)		*p*
**Age**	**Mean ± SD**	45.52 ± 10.8		39.02 ± 11.11		42.44 ± 11.92		0.121 *
**Gender**	**Male**	13	48.15%	13	52.00%	14	56.00%	0.852 †
	**Female**	14	51.85%	12	48.00%	11	44.00%	
**Lesion side**	**Right**	12	44.44%	12	48.00%	12	48.00%	0.956 †
	**Left**	15	55.56%	13	52.00%	13	52.00%	
**Dominant extremity**	**Right**	19	70.37%	16	64.00%	15	60.00%	0.731 †
	**Left**	8	29.63%	9	36.00%	10	40.00%	
**BMI**	**Mean ± SD**	29.7 ± 4.22		29.08 ± 4.63		29.47 ± 4.66		0.881 *
**Smoking**	**−**	15	55.56%	16	64.00%	16	64.00%	0.769 †
	**+**	12	44.44%	9	36.00%	9	36.00%	
**Smoking (package/day/year)**	**Mean ± SD**	14.5 ± 7.94		11.33 ± 8.05		7.89 ± 4.17		0.124 *
**Symptom duration (months)**	**Mean ± SD**	18.22 ± 8.15		18.24 ± 13.96		24.68 ± 23.77		0.336 †
	**Median (IQR)**	16 (12–24)		12 (10.5–21)		15 (12–35.5)		

* One-way analysis of variance, † Kruskal–Wallis test, SD: standard deviation, IQR: interquartile range, BMI: body mass index.

**Table 2:** Comparison of AOFAS and VAS scores of microfracture, nanofracture, and K-wire drilling groups.

		Microfracture (*n*: 27)		Nanofracture (*n*: 25)	Drilling (*n*: 25)		*p* *
**AOFAS**	**preop**	43.63 ± 12.01		49.84 ± 14.47	52.32 ± 14.48		0.053
	**6th month**	75.63 ± 9.75		74.8 ± 11.71	75.2 ± 8.08		0.956
	**12th month**	93.48 ± 4.8		94.24 ± 5.56	95.08 ± 4.84		0.528
	***p* ‡**	**0.0001**		**0.0001**	**0.0001**		
**VAS**	**preop**	6.89 ± 1.34		6.92 ± 1.63	6.04 ± 1.51		0.066
	**6th month**	3.41 ± 1.01		3.72 ± 1.37	3.12 ± 0.97		0.178
	**12th month**	1.07 ± 0.96		1.28 ± 1.28	0.72 ± 0.54		0.127
	***p* ‡**	**0.0001**		**0.0001**	**0.0001**		
**Newman–Keuls Multiple Comparison Test**		**AOFAS**			**VAS**		
		**Microfracture**	**Nanofracture**	**Drilling**	**Microfracture**	**Nanofracture**	**Drilling**
**preop/6th month**		**0.0001**	**0.0001**	**0.0001**	**0.0001**	**0.0001**	**0.0001**
**preop/12th month**		**0.0001**	**0.0001**	**0.0001**	**0.0001**	**0.0001**	**0.0001**
**6th month/12th month**		**0.0001**	**0.0001**	**0.0001**	**0.0001**	**0.0001**	**0.0001**

* One-way analysis of variance, ‡ paired analysis of variance, AOFAS; American Orthopedic Foot & Ankle Society score, VAS; visual analogue scale score. Values in bold indicate statistically significant difference.

**Table 3:** Comparison of % change difference values of AOFAS and VAS scores of microfracture, nanofracture, and K-wire drilling groups.

% Change Difference Value		Microfracture (*n*: 27)	Nanofracture (*n*: 25)	Drilling (*n*: 25)	*p* *
**AOFAS**	**preop–6th month**	43.28 ± 15.04	33.17 ± 16.13	30.86 ± 15.66	**0.012**
	**preop–12th month**	19.14 ± 9.32	20.77 ± 10.2	20.91 ± 7.46	0.734
**VAS**	**preop–6th month**	50.56 ± 11.07	46.11 ± 15.71	47.94 ± 11.44	0.458
	**preop–12th month**	84.97 ± 12.2	82.84 ± 16.24	88.35 ± 9.15	0.316
**Tukey’s Multiple Comparison Test**			**AOFAS 6th Month Preop**		
**Microfracture/Nanofracture**			**0.047**		
**Microfracture/Drilling**			**0.015**		
**Nanofracture/Drilling**			0.850		

* One-way analysis of variance, AOFAS; American Orthopedic Foot & Ankle Society score, VAS; visual analogue scale score. Values in bold indicate statistically significant difference.

## Data Availability

The original contributions presented in this study are included in the article. Further inquiries can be directed to the corresponding author.

## Abbreviations

The following abbreviations are used in this manuscript:

AOFAS	American Orthopedic Foot & Ankle Society score
VAS	Visual analogue scale score
AAS	Ankle arthritis score
BMI	Body mass index
CT	Computed tomography
K	Kirschner wire
